# Diagnosing gait disorders based on angular variations of knee and ankle joints utilizing a developed wearable motion sensor

**DOI:** 10.1049/htl2.12015

**Published:** 2021-06-14

**Authors:** Ardalan Akhavanhezaveh, Reza Abbasi‐Kesbi

**Affiliations:** ^1^ Faculty of Engineering Monash University Melbourne Australia; ^2^ MEMS & NEMS Department Faculty of New Sciences and Technologies University of Tehran Tehran Iran

## Abstract

Here, a sensory motion system is developed to diagnose gait disorders using the estimation of angular variations in the knee and ankle joints. The sensory system includes two transmitter sensors and a central node, where each transmitter comprises three sensors of accelerometer, gyroscopes, and magnetometer to estimate the angular movements in the ankle and knee joints. By using a proposed filter, the angular variation is estimated in a personal computer employing the raw data of the motion sensors that are sent by the central node. The obtained results of the presented filter in comparison to an actual reference illustrate that the root mean square error is less than 1.01, 1.34, and 1.61 degrees, respectively, for the angles of ϕ and θ and ψ that illustrate an improvement of 40% than the previous work. Moreover, a quantity value is defined based on the correlation between knee and ankle angles that show the amount of correctness in gating. Thus, the proposed system can be utilized for people who suffer problems in gait and help them to improve their movements.

## 1 INTRODUCTION

Gaiting is a substantial need of every person to move from one place to another, and for this purpose, the foot is one of the essential organs in the human body [1]. Two‐legged movement involves a cycle of activities that has two stages of swinging and stance for each lower limb. Gait is symmetrical action due to the angular movements of the main joints, the pattern of muscle activity, weight‐bearing on the lower limbs, and generally the transfer of the body's center of gravity [2]. A complete gait cycle is defined as the successive occurrence of the swing and stance stages by one foot [3, 4]. Each of the two gaiting steps consists of several sub‐sections. A complete gaiting cycle is called a stride, which begins with the occurrence of one of the sub‐sections of each of the two steps, and one stride is completed when it reaches the same sub‐section with the same foot [5]. For example, a complete gait cycle or one stride is called from the contact of the heel of one foot with the ground to the next contact of the same heel that the stride forms 60% of a stance. This stage lasts from the beginning of the contact of one foot with the ground until the separation of the same foot. In this stage, the foot bears the weight of the body. Moreover, the stride makes 40% of a swing that is as a stage where part of the foot is in the air [6, 7].

Biomechanically, the lower limb should distribute the flexural, torsional, shear, and compressive forces well in the stance phase of gait [8]. Improper distribution of these forces may cause abnormal movement and, as a result, an additional load on the tissues of the foot, thereby causing soft tissue damage and muscle dysfunction [9, 10]. When a person suffers from a gait disorder, it takes a long time to recognize the problem because this issue requires expert opinions in this field. Additionally, the person should visit the clinic frequently and perform gait analyses to solve the drawback [11]. The therapists first analyse the patients' gait in clinics by observing and evaluating a normal stride and gait. Then, they determine a treatment plan for patients based on disorders caused by age, speed, slopes, steps, and abnormal effects such as weakness, seizures, deformities, and pain in which can significantly affect typical functioning [12, 13, 14].

The need for gait knowledge can help to accurately assess and examine other patient problems such as involvement of the nervous and musculoskeletal systems. Accordingly, the physiotherapist can be one of the best therapists in this field with having this knowledge [15]. However, gait and stride disorders can be corrected and repaired either by a physiotherapist and surgeon or by artificial limbs or orthoses. The physiotherapist evaluates the spatio‐temporal parameters of gait, step kinematic measurement, three‐dimensional step analysis, standing biomechanics, ground reaction force in usual gaiting, measurement of sole pressure, joint torque, muscles, pathological gait analysis, and other parameters [16, 17]. Finally, they help the patients by giving the right training, corrective exercise, providing the correct gaiting pattern besides treating the underlying cause of the disorder. The issue of patients' movement and gaiting is more significant for physiotherapists and therapists that an essential part of treatment accounts for bringing people back into the community. The movement recognizing and evaluating as an initial point is significant for starting treatment and rehabilitation [18, 19]. Additionally, the foot kinematic has an essential role in the gaiting study. For example, the effect of soft tissue on foot kinematic in gaiting was investigated in [20] that used many markers for this purpose. Their result revealed that the soft tissue artifacts were the most effective on joint angles in the sagittal plane for Rizzoli foot models and the other two planes for Oxford foot models [20]. In another study, a system was developed for foot kinematic based on pressure and EMG sensor for gaiting [21]. Nonetheless, their system was bulky and non‐wireless. Daily human kinematic gait analysis was investigated by a wearable inertial sensor system in [22]. However, their system is not very accurate and they used two sensor gyroscopes and accelerometer.

Although these gait analyses can be used to diagnose gait problems, their equipment is adequately expensive and require massive set up to measure. Besides, patients have to spend a lot of time on these analyses, and can be tedious for them because of going to the clinic, getting tests, and analysing. All of the processes are very time‐consuming [23, 24]. Other systems were introduced to solve the mentioned problems which are called inertial systems [25, 26, 27]. These systems are cheaper and can be easily used everywhere, especially at home. Many inertial systems have been used for lower‐limb monitoring. In one study in [28], accelerometer and gyroscope sensors were used to measure foot angle, however, the accuracy of their system was low due to the lack of a magnetometer sensor. Furthermore, a system was developed for foot monitoring so that the angles of the foot can be recorded in different positions [29]. Nonetheless, their system was bulky as well as their accuracy in estimation of joint angles was not very high. In addition, they sent the data with the wire that it made some stress for patients when they were testing. An accelerometer and gyroscope sensors were used along with magnetic sensors to detect various types of motion in [30]. One of the problems in their work was a large number of devices for estimating the types of movements.

Here, a miniature motion system is developed to estimate joint angles of the knee and ankle for foot monitoring and diagnostic of gait problems. For this purpose, first, a small printed circuit board is developed for collecting the raw data, and then a complementary filter is proposed to estimate angular movement and eliminate stochastic and bias errors. The developed sensory system is tested and compared with a reference that the obtained results of the proposed filter reveal that accuracy improves by 40% compared to previous examples of angle estimation in [29, 31, 32, 33]. Then, the developed system is mounted on the ankle and knee of a volunteer and gait problems are diagnosed by taking a correlation between the angular output of the ankle and knee. The results demonstrate that there is a correlation of less than 0.42 for healthy gaiting while this value is more than 0.5 for a patient.

## 2 METHODOLOGY

The main purpose of the paper is to diagnose gait disorders using a developed miniature sensor. To this end, first, a motion sensor is developed that estimates the ankle and knee angles based on a proposed filter. Then, the correlation between the ankle and knee angles in gaiting is calculated and the rate of improvement is defined based on a threshold that is extracted using the data of several volunteers.

### 2.1 Angle estimation

An accelerometer, a gyroscope, and a magnetometer are used to estimate the angular movements (Euler angles) [34]. However, the angular movements cannot be estimated by one of the mentioned sensors alone because there is a deviation in the output of the sensors [35]. For example, gyroscope data is valid for short periods and deviates over long periods. Also, accelerometer data is reliable in the long run [36]. A combination of accelerometer and gyroscope sensors can be used to estimate accurate roll (ϕ) and pitch (θ) angles. Nonetheless, the fusion of the two sensors is not enough to achieve precise angle movements in yaw (ψ) angles [37, 38]. Thus, a magnetometer sensor is added to the two accelerometer and gyroscope sensors to improve the angle of yaw. Accordingly, Equation (1) is defined as follows to fusion accelerometer, gyroscope, and magnetometer data:

(1) $${\dot{q}}_{n,t}={\dot{q}}_{g,t-1}-\lambda \left({\dot{q}}_{m,t-1}+{\dot{q}}_{a,t-1}\right).$$

Where ${\dot{q}}_{g,t-1}$ is the quaternion derivative of the gyroscope data at time *t* ‐ 1, ${\dot{q}}_{m,t-1}$ is the quaternion derivative of the magnetometer data, and ${\dot{q}}_{a,t-1}$ is the quaternion derivative of the accelerometer data. It is quite clear that the accelerometer and magnetometer data are added together and multiplied by λ, which is the attenuation coefficient, and finally reduced from the gyroscope data to eliminate the amount of deviation. The gyroscope sensor records three‐dimensional angular velocity. In order to convert the three‐dimensional angle to quaternion derivative Equation (2) is used [39]:

(2) $${\dot{q}}_{g,t}=\frac{1}{2}q_{n,t-1}⊗\left(w_{r,t}-E_{bg}\right).$$

Where $w_{r,t}$ is the raw data of the gyroscope, $E_{bg}$ is the bias of the gyroscope, and $q_{n,t-1}$ is the quaternion data in the previous period. It should be noted that the initial value of the gyroscope sensor is averaged in the range of 50 to 100 samples to eliminate the bias error, and then subtracted from the raw data. In the following, the accelerometer and magnetometer meter data is converted to quaternion derivative [40, 41]. To this purpose, first, the following equation is defined to eliminate gravitational acceleration:

(3) $$a_{u,t}=a_{r,t}-a_{g,t}.$$

Where $w_{r,t}$ is the raw data of the gyroscope, $E_{bg}$ is the bias of the gyroscope, and $q_{n,t-1}$ is the quaternion data in the previous period. It should be noted that the initial value of the gyroscope sensor is averaged in the range of 50 to 100 samples to eliminate the bias error, and then subtracted from the raw data. In the following, the accelerometer and magnetometer data is converted to quaternion derivative. To this purpose, first, the following equation is defined to eliminate gravitational acceleration:

(4) $$a_{g,t}=M_{r,t-1}\left[\begin{array}{c} 1\\ 0\\ 0 \end{array}\right].$$

Where $M_{r,t-1}$ is a rotational matrix and is defined as Equation (5) [42].

(5) $$M_{r}=\left[\begin{array}{ccc} q_{1}^{2}+q_{2}^{2}-0.5 & q_{2}q_{3}+q_{1}q_{4} & q_{2}q_{4}-q_{1}q_{3}\\ q_{2}q_{3}-q_{1}q_{4} & q_{1}^{2}+q_{3}^{2}-0.5 & q_{3}q_{4}+q_{1}q_{2}\\ q_{2}q_{4}+q_{1}q_{3} & q_{3}q_{4}-q_{1}q_{2} & q_{1}^{2}+q_{4}^{2}-0.5 \end{array}\right].$$

The Earth's magnetic field can be considered as two vertical and horizontal vectors. The magnetometer sensors suffer deviations which is called hard and soft iron. First, the magnetometer data is modeled as follows:

(6) $$m_{u,t}=m_{r,t}-s_{i,t}-H_{i,t}.$$

Where, $m_{u,t}$ is new magnetometer data, $m_{r,t}$ is raw magnetometer data, $s_{i,t}$ is soft iron effect and $H_{i,t}$ is hard iron effect. The soft iron effect is defined as Equation (7):

(7) $$s_{i,t}=M_{r,t-1}\left[\begin{array}{c} \sqrt{h_{x,t}^{2}+h_{y,t}^{2}}\\ 0\\ h_{z,t} \end{array}\right].$$

Where h is the compass data after removing the hard iron effect and multiplying in rotational matrix [42], as shown in Equation (8):

(8) $$\left[\begin{array}{c} h_{x,t}\\ h_{y,t}\\ h_{z,t} \end{array}\right]=M_{r,t-1}\left[\begin{array}{c} m_{r,x,t}-H_{i,x,t}\\ m_{r,y,t}-H_{i,y,t}\\ m_{r,z,t}-H_{i,z,t} \end{array}\right].$$

Now, Equation (9) is defined to convert the accelerometer and magnetometer quaternion data to their quaternion derivative, that $J_{m}$ and $J_{a}$ are Jacobian matrices of magnetometer and accelerometer [43, 44], and their values are as follows:

(9) $${\dot{q}}_{m,t}+{\dot{q}}_{a,t}=J_{m}m_{u,t}{{|}_{J_{m}=\frac{ds_{i,t}}{dq}}+J_{a}a_{u,t}|}_{J_{a}=\frac{da_{g,t}}{dq}}.$$

Finally, the value of the new quaternion is obtained by integration of the quaternion derivative:

(10) $$q_{n,t}=q_{n,t-1}+{\dot{q}}_{n,t}▵t.$$

Where ▵t is the data sampling rate. Then, the quaternion data is converted to Euler angles by Equation (11) to record the angular variation of the knee and ankle.

(11) $$\begin{array}{ccc} \phi & = & tan^{-1}\left(\frac{q_{2}q_{3}-q_{0}q_{1}}{q_{0}^{2}+q_{3}^{2}-0.5}\right),\\ \theta & = & -sin^{-1}\left(2q_{1}q_{3}+2q_{0}q_{2}\right),\\ \psi & = & tan^{-1}\left(\frac{q_{1}q_{2}-q_{0}q_{3}}{q_{0}^{2}+q_{1}^{2}-0.5)}\right). \end{array}$$

After obtaining the angle values, the correlation of the two ankle and knee angles is measured by Equation (12). If the correlation is more than 0.5, it can be said that the foot does not move properly. However, if the correlation is less than 0.5, the foot angles work almost correctly in gaiting.

(12) $$q_{f}=Corr\left(E_{a},E_{k}\right).$$

Where $E_{a}$ and $E_{k}$ are Euler angles of ankle and knee joints, respectively. Moreover, Euler angles consist of three angles of ϕ, θ, and ψ.

### 2.2 The developed system

**FIGURE 1:**
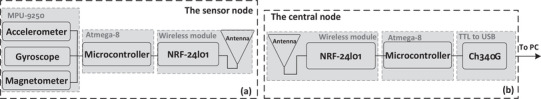
The schematic of the developed system: (a) transmitter and (b) the central node

The proposed system, which estimates joint angles in the knee and ankle, includes two transmitters and one central node. Each of the transmitter sensors consists of an MPU‐9250 [45], an NRF24L01 [46], and an Atmega8 [47], as shown in Figure 1a. These transmitters are supplied by a lightweight lithium polymer battery of 3.7 V, 380 mAh. The MPU‐9250, NRF24L01, and Atmega8 have low power consumption, are low priced, and are very common in the market.

The MPU‐9250 sensor is an improved version of the MPU‐9150 in a smaller chip with less power consumption [45]. This sensor includes three sensors of 3D accelerometer, 3D gyroscope, and 3D magnetometer. The variation ranges of the gyroscope can be in ±250, ±500, ±1000, and ± 2000 degrees per second. The sampling rate of the analog to digital converter can be programmed from 3.9 samples per second to 8000 samples per second [45]. There is also a low‐pass filter in this area that can be adjusted in a wide range. The range for accelerometers is ± 2g, ±4g,±8g, ±16g. The analog‐to‐digital converter is 16‐bit for the accelerometer and gyroscope [45]. Nevertheless, the compass used in this sensor has a 13‐bit analog‐to‐digital converter. The maximum measurable range of this compass can be between ±1200 microtesla. The MPU‐9250 communicates with the processor via the $I_{2}C$ interface and sends all its information to a processor through this interface [45].

It may be said that any module can be used to transmit the obtained data wirelessly, but it is not true because one of the significant factors in the proposed study is being small. Another essential feature is the bit rate of data transferring to record every movement accurately. If the bit rate of data is low, the foot movements will not be simulated naturally. Another essential characteristic is the cost of this module. According to these features, an NRF24L01 module is used. The wireless module created an impressive improvement in power efficiency for a one‐way and a two‐way system, without adding complexity to the program [46]. As a result, power consumption is adequately low.

In the processor, factors such as small size, low power consumption, low cost, and flash memory are essential. According to the available processors in the market, an SMD microcontroller of Atmega8 with AVR series is employed. This processor has the smallest size among Atmegas and has 8 KB of flash memory, 512 bytes of EEPROM memory, 2 kilobytes of SRAM, 6 of analog‐to‐digital converter of 10‐bit, 32 input and output ports, SPI connection, USART connection, serial two‐wire connection (UART), internal and external interrupts, and three timers with operating voltage between 2.7 to 5.5 V [47]. Their frequency is from 1 to 16 MHz, and the power consumption of this processor is adequately low, which makes it suitable for this purpose.

**FIGURE 2:**
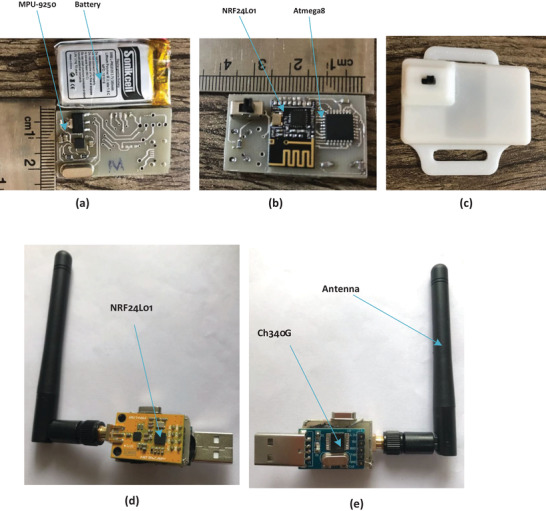
The prototype of the developed transmitter: (a) top layer and battery, (b) bottom layer, and (c) all of the components in a developed box. The prototype of the central node: (d) top layer and (e) bottom layer

The mentioned microcontroller processes the data in both the transmitter and the receiver. The transmitters include a microcontroller of Atmega8, an MPU‐9250, and an NRF24L01 shown in Figure 2a–c. The data of MPU‐9250 is read by the microcontroller via the I2C protocol and then is transmitted to the NRF24L01 through the SPI protocol. Initial registers are defined in the transmitter in Atmega8 to read data from the MPU‐9250 and send it to the NRF24L01. For this purpose, these registers are defined based on data sheets in [45, 46]. Accordingly, the first address of the register is sent by Atmega8 on the I2C bus to read the data of MPU‐9250, and then the value of the address is put back on the bus by the processor of MPU‐9250, and finally, Atmega8 receives the data. In order to send the data to NRF24L01, some registers are defined in Atmega8 and after receiving the data MPU‐9150 by Atmega8, it is sent to NRF24L01.

The central node consists of an Atmega8, an NRF24L01, and a TTL to USB converter (Figures 1b and 2c,d), and the data is briefly taken by NRF24L01 using the Atmega8 and then sent to the serial port of a personal computer. Indeed, the initial registers should be defined in Atmega8 of the central node for receiving the data and correct communication with NRF24L01. These registers include specifying the transmitter and receiver address, specifying the address of each byte of transmitted information, determining the transmitter and receiver operating frequency, operating bandwidth, and bit rate of data, and acknowledgment [46], [48]. These registers are also defined in the transmitter for communication between Atmega8 and NRF24L01. After sending a few bytes of data by the transmitter, the data is received by the microcontroller (Atmega8) using NRF24L01 via the SPI bus. Then, the microcontroller sends it to the USB port of a personal computer through the UART protocol to display and perform complex mathematical operations that cannot be written in the microcontroller. The data is sent at a rate of 115,200 bits per second to the computer serial port. The bit rate is maximum for sending data through the serial port and helps to accurate monitoring of movements. According to the transmitted data of the accelerometer, gyroscope, and magnetometer, the sample rate is 50 Hz.

## 3 RESULTS AND DISCUSSIONS

In order to more clarify, the experimental protocols are defined as follows: First, the accuracy of the developed system is investigated by a computer numerical control device (CNC), and then some volunteers are asked to mount the developed system on the knee and ankle and walk in a path while the system records the angular estimation of knee and ankle. By taking the correlation between the knee and the ankle, a threshold is obtained that can help to diagnose the disorder in the gaiting analysis.

### 3.1 Accuracy in the developed system

**FIGURE 3:**
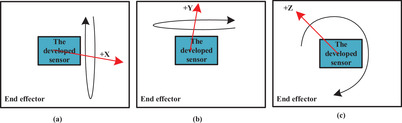
Schematic representation for the assessment of the sensor accuracy. The rotation of (a) around *x*‐axis, (b) *y*‐axis, and (x) *z*‐axis

To determine the accuracy of the developed sensor, the estimated angles in the system are compared to actual angles. To this end, a CNC is used as the reference (actual value) whose accuracy is 0.001 degrees. First, this sensor is fixed on the end effector of the CNC using strong tape and standard plastic clamps (Figure 3). Then, various commands are given to CNC to rotate its end effector at different angles. At the same time, both the output data of CNC and the developed sensor are recorded through the input port of the personal computer and then compared. Three movements around three axes of x,y,andz were performed in a time of approximately 15 min while the developed sensor was turned by the CNC. Initially, only the *x*‐axis is rotated at different angles by CNC. In the next part, the rotation around the *y*‐axis and finally, the rotation around the *z*‐axis are examined at different angles, as shown in Figure 3. Finally, the obtained angles are estimated using the proposed filter and compared with the reference.

**FIGURE 4:**
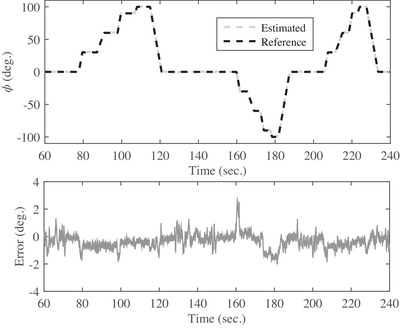
The snapshot of the obtained results of angular variations between the proposed filter and CNC around *x*‐axis

**FIGURE 5:**
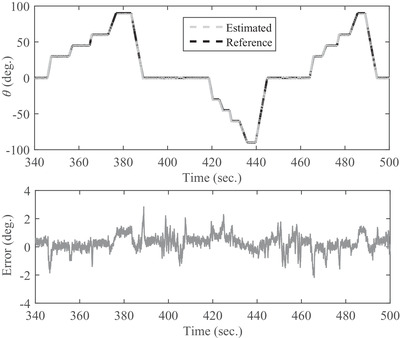
The snapshot of the obtained results of angular variations between the proposed filter and CNC around the *y*‐axis

**FIGURE 6:**
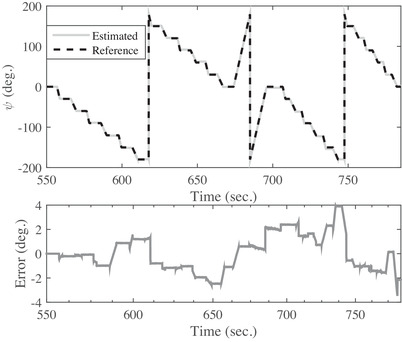
The snapshot of the obtained results of angular variations between the proposed filter and CNC around the *z*‐axis

The output of the developed system using the proposed filter in three rotation modes around axes of x,y, and z is shown in Figures 4, 5, and 6. The obtained results of the estimated and actual angles for ϕ angle are illustrated in Figure 4. The first test is performed with a duration of 270 s around the *x*‐axis for all angles consist of 45, 90, 130, 150, and 180 degrees. Also, the maximum error is 3.7 degrees and the root mean square error is obtained less than 1.01 degrees for the angles of ϕ, as shown in Figure 4 and Table 1, respectively. In the next test, the CNC rotates all over the direction from ‐90 to 90 degrees in the next 270 s for θ angels and a snapshot of the obtained results for angular movements shown in Figure 5. As the outcome results show, the maximum error is about 2.8 degrees. Also, the root mean square error is obtained less than 1.34 degrees and is shown in Table 1. In the last test to measure the accuracy of the developed sensor, the CNC rotate only in the various angle of the *z*‐axis from time of 570 to 810 s. The obtained results in Figure 6 reveals the maximum error is 3.9 degrees, and the root mean square error is less than 1.61 degrees for ψ angle. Briefly, the root mean square error of the presented filter in comparison with the actual value is shown in Table 1. As Table 1 shows, there are two dynamic and static errors. The dynamic error is defined when the sensor has angular variations than the previous states while the static error happens when the sensor is constant. In other words, the dynamic error is the difference between the actual and estimated angles when the sensor is moving, and the static error is related to the error of the actual and estimated angles when the sensor is without any movement.

**TABLE 1:** Root mean square error of the presented filter

Error (${}^{\circ }$)	Static	Dynamic
ϕ	0.8	1.01
θ	1.07	1.34
ψ	1.56	1.61

As previously mentioned, λ is called the convergence coefficient in Equation (1), and its value is between 0 and 1. This value increases or decreases the effect of accelerometer and magnetometer data fusion on the gyroscope data. If it is selected incorrectly, the amount of extra jump is high in changing from one angle to another. Also, output oscillation occurs when the sensor is constant, and its stability reduces. The best λ is considered 0.04 in the proposed filter. Additionally, ▵t is considered 20 ms in Equation (10) because the sample rate of recoding movements is 50 Hz, and it means that a sample of motion is recorded approximately every 20 ms.

As the obtained result reveal, the accuracy of the proposed filters is better than [29, 31, 32, 33, 49] for all three angles. In reference [29], the static angle error was less than 2.5 degrees and the dynamic angle error was less than 3.5 degrees and also, a mean RMSE of 3.3 degrees was reported in [31]. Moreover, a system for monitoring lower‐limb joints was developed in [32] that its error was less than 3.38 degrees. Accordingly, at least an improvement of 40% is done in error reduction for the proposed filter in this paper. Moreover, although some works are observed in the estimation of joint angles, there are some differences between the proposed system and them. For example, filters were used in [50] that are different from the proposed filters. Here, a filter is proposed that works based on a descending gradient and is in quaternion space. However, the filter in [50] works with the help of three variables and not quaternion. Besides, the proposed system benefits three sensors of accelerometer, gyroscope, and magnetometer despite the work in [50] which used two accelerometer sensors and a gyroscope for estimation of joint angles. As mentioned earlier in the methodology section, good accuracy is not obtained with the help of two sensors of gyroscope and accelerometer, because the gyroscope deviations cannot be eliminated well with the help of an accelerometer.

### 3.2 Diagnosing gait disorders

**FIGURE 7:**
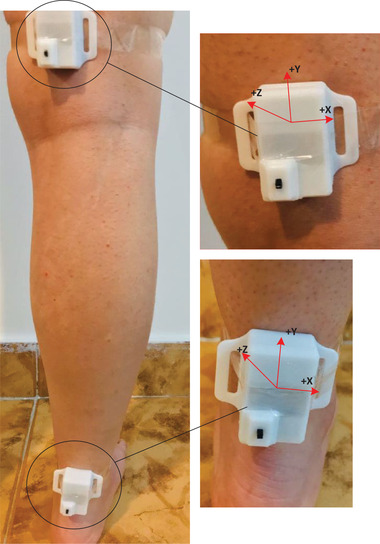
The location of the developed sensors on knee and ankle joints to measure angular movements

First, the measurement setup shown in Figure 2 comprises one central node and two transmitters. The transmitter is developed to be as small as a fingertip and is smaller than other works in [31, 51, 52]. The developed system's size is 35 mm × 22 mm × 10 mm (Figure 2a–c) to help volunteers in performing their work without restrictions. Another outstanding feature of this circuit is its low power consumption of 60 mW, and it can easily last up to 15 h with a lithium polymer battery 3.7 V 380 mA. With the help of the proposed system, acceleration, angular velocity, and magnetic field are measured in the direction of the three coordinate axes and sent to the serial port of the computer for further investigation and angle estimation. In comparison to other industrial wireless angle systems in [53, 54, 55, 56], the developed system has a smaller size, less power, and less weight (10 g). To carry out the experiments, the two developed sensors are connected to the knee and ankle joints of volunteers, as shown in Figure 7, and the central node is connected to the USB port of a personal computer. Table 2 shows the volunteers' characteristics such as age, weight, height, and gender. As can be seen, volunteers with different characteristics have been incorporated into the experiments. Moreover, the volunteers first have to stand for 2–5 s and then move their feet. It should be noted that the sensor should be fixed on their joints strongly. If the sensor is not fixed on the joint tightly, the results are not acceptable.

**TABLE 2:** The volunteers characteristics in the mentioned experiments

Volunteer	Age	Weight (kg)	Height (cm)	Gender
1	50	71	179	Male
2	43	69	160	Female
3	28	54	165	Female

**TABLE 3:** Correlation of angular variations for knee and ankle joints

Volunteer	$q_{f,\phi }$	$q_{f,\theta }$	$q_{f,\psi }$	mean
1	0.93	0.61	0.85	0.80
2	0.97	0.85	0.90	0.91
3	0.27	0.51	0.50	0.43

**FIGURE 8:**
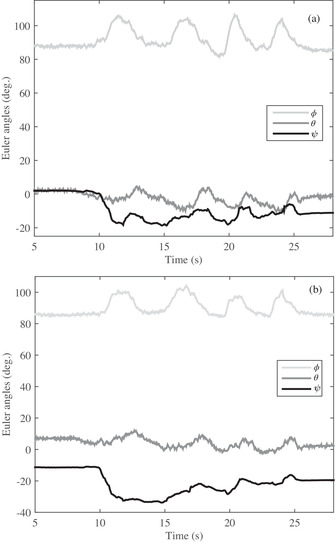
The angular variation of the developed sensors for volunteer#1: (a) ankle and (b) knee

**FIGURE 9:**
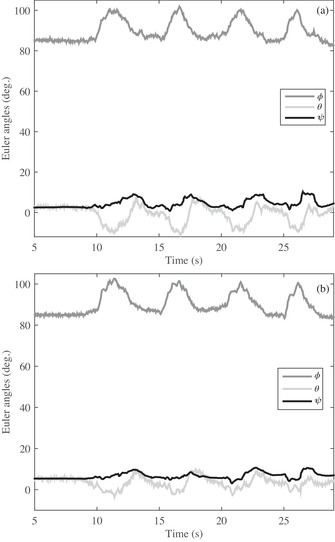
The angular variation of the developed sensors for volunteer#2: (a) ankle and (b) knee

**FIGURE 10:**
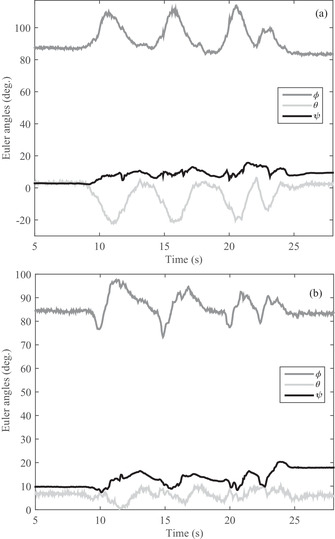
The angular variation of the developed sensors for volunteer#3: (a) ankle and (b) knee

In the first test, a volunteer who has a problem in gaiting is asked to gait after mounting the developed sensor on his ankle and knee. The angular variations of three angles of ϕ, θ, and ψ in knee and knee joints are observed in Figure 8a,b, in turn; the two (Figure 8a,b) are almost identical. It should be noted that the two sensors are initially positioned at an angle of approximately 90 degrees of θ angle, so the initial value of both sensors starts at approximately 90 degrees. As shown in Figure 8, the volunteer moves his foot four steps in a time of 31 s so that the changes are easily visible. Also, the steps are visible not only for θ angle but also for the ϕ and ψ angles. In another test, another patient is asked to gait while the proposed system has been attached to her knee and ankle. The obtained results of angle output for the knee and ankle is shown in Figure 9a,b. As Figure 9 shows, the angular variations of the knee and ankle are similar around the *x*‐axis (ϕ) because the patient moved the knee and ankle together at the same angle, which indicates a movement defect. Furthermore, the θ and ψ for knee and ankle angles resemble together in Figure 9a,b. In the last test, a healthy person is asked to gait with the developed sensor. The obtained results in Figure 10a,b illustrate that the angular variations around the *x*‐axis are different for ankle and knee despite patients in Figures 8 and 9. Indeed, the healthy person moved their knee and ankle at different angles simultaneously, while patients pull their foot on the ground, and the knee and ankle are not changed differently. Thus, the outcome results of the Euler angles of the knee and ankle joint are similar for the patients while the value is different for the healthy person. Additionally, the angular variations not only are different around the *x*‐axis for a healthy person but also in the other axes of the *y*‐axis and *z*‐axis. Nevertheless, the knee and ankle angles are similar for patients who are incorporated in the tests.

Now, the output of the angle of each test is separately investigated in terms of correlation by Equation (12) and their results are shown in Table 3. The first volunteer (volunteer#1), who cannot properly move his knee and ankle together, there is a high correlation between the knee and the ankle, and its value is 0.93, 0.61, and 0.85 in angles of ϕ, θ, and ψ, separately. In the next volunteer (volunteer#2), when the patient is unable to move his foot properly and pull his foot on the ground, the correlation is obtained as 0.97, 0.85, and 0.90 for ϕ, θ, and ψ angels, separately, although a little more than the previous volunteer. The last volunteer (volunteer#3), who correctly moves his foot in gaiting, the correlation between knee and wrist is obtained as 0.27, 0.51, and 0.50 for angular variation of ϕ, θ, and ψ, in turn. All the results are shown in Table 2. It should be noted, the correlation of every axis is separately calculated and gained an average correlation for volunteers. As Table 2 shows, the mean values are 0.80, 0.91, and 0.43, respectively, for these three volunteers. If the value is less than 0.5, volunteers carry out proper gaining. Nonetheless, if the value is more than 0.5, the gaiting is not appropriate. Therefore, this system can give a quantity to patients who suffer gait problem and assist to improve their walk until reaching a balancing gait.

## 4 CONCLUSIONS

Here, a sensory system for estimation of the angular variation of knee and ankle joints was developed to improve gaiting in patients. This developed sensor is so small as that volunteers can mount it in their joints and easily gait. Furthermore, a filter was proposed to estimate the angular variations in knee and ankle joints that the obtained results proved an improvement of 40% than other studies. The developed system was used by several healthy and unhealthy volunteers to measure angular variation in their knee and ankle. The obtained results showed that the angular changes of the knee and ankle are different for a healthy person, and its correlation acquired less than 0.5 while the angular variations of knee and ankle for patients are similar together, and its correlation was more than 0.5. Therefore, patients who experience drawbacks in gaiting can benefit from the proposed system to improve their gaiting properly.
